# Factors influencing participation and long-term commitment to self-monitoring of blood pressure in a large remote clinical trial: The treatment in morning versus evening (TIME) study

**DOI:** 10.1038/s41371-021-00621-5

**Published:** 2021-10-19

**Authors:** Keeran Vickneson, Amy Rogers, Thineskrishna Anbarasan, David A. Rorie, Thomas M. MacDonald, Isla S. Mackenzie

**Affiliations:** 1 Medical Student, Medicines Monitoring Unit (MEMO Research), Division of Molecular and Clinical Medicine, University of Dundee, Dundee, UK; 2 Clinical Research Fellow, Medicines Monitoring Unit (MEMO Research), Division of Molecular and Clinical Medicine, University of Dundee, Dundee, UK; 3 BHF Senior Software Developer, Medicines Monitoring Unit (MEMO Research), Division of Molecular and Clinical Medicine, University of Dundee, Dundee, UK; 4 Medicines Monitoring Unit (MEMO Research), Division of Molecular and Clinical Medicine, University of Dundee, Dundee, UK

**Keywords:** Diagnosis, Clinical trials, Risk factors

## Abstract

This study investigates factors associated with active participation, and long-term commitment, to home blood pressure monitoring (HBPM) in the TIME study, a remote clinical trial assessing the effectiveness of morning vs. evening dosing of antihypertensive medications on cardiovascular outcomes in adults with hypertension. Participants reporting HBPM ownership were invited to submit blood pressure (BP) measurements three-monthly. Factors associated with active participation (submitting at least one set of BP measurements), and longer-term commitment (at least six sets of BP measurements), were analysed using multivariable logistic regression. 11,059 participants agreed to provide BP measurements, of whom 7646 submitted. Active participation was associated with age (adjusted odds ratio (AOR) per decade, 1.29; 95% CI 1.23–1.36), positive family history of hypertension (AOR 1.11; 95% CI 1.01–1.21), number of antihypertensive medications (AOR, 1.10; 95% CI 1.04–1.16), and lower deprivation (AOR per decile, 1.03; 95% CI 1.01–1.05). People with higher body mass index (BMI) and smokers were less likely to participate (AOR, 0.91 (per increase of 5.0 kg/m^2^) and 0.63 respectively; all *p* < 0.001). 3,655 participants (47.8%) submitted measurements beyond one year. Non-modifiable risk factors – age (AOR per decade, 1.29; 95% CI 1.21–1.37) and positive family history of hypertension (AOR, 1.15; 95% CI 1.03–1.27) – were positively associated with longer-term commitment. Higher BMI (AOR per 5.0 kg/m^2^, 0.89; 95% CI 0.85–0.93), smoking (AOR 0.60, 95% CI 0.44–0.82) and higher baseline systolic blood pressure (AOR per mmHg, 0.99; 95% CI 0.98–0.99) were negatively associated. This study provides insight into factors that influence HBPM use.

## Introduction

Hypertension is a major risk factor for cardiovascular disease and is the largest contributor to cardiovascular morbidity and mortality worldwide [1, 2]. In the UK, control of hypertension in the community has improved but remains sub-optimal despite greater awareness and wide availability of effective treatment [3, 4]. One approach to bridging this gap is encouraging more active involvement of patients in monitoring and managing their hypertension with appropriate guidance from health professionals. Trials of self-monitoring and hypertension management using home blood pressure monitors (HBPMs) have shown significant benefits in reducing elevated blood pressures [5, 6]. Home blood pressure measurements are more reliable and more strongly correlated with long-term complications of hypertension than office-based measures [7].

Randomized controlled trials provide robust evidence on the safety and clinical benefit of interventions [8]. However, the limited external validity of trials is a frequent criticism [9, 10]. Patients with hypertension are more willing to participate in antihypertensive drug trials if they are younger, non-smokers, or have previously participated in research [11]. However, the influence of baseline demographics on the likelihood of participating in a remote clinical trial is unknown. In theory, remote studies should improve external validity by widening access and may encourage participant retention by removing the burden of physical face-to-face study visits [12–14].

The TIME study is an ongoing, prospective, randomized, open-label, blinded end-point (PROBE) design clinical trial investigating the effect of morning versus evening dosing of antihypertensive medications on cardiovascular outcomes in 21,104 participants with hypertension [13]. The study is conducted online via a secure website. Study participants who reported owning an HBPM at study entry were invited to submit BP measurements throughout the study. In the UK, the use of HBPMs is increasing, with ~30–40% of people with hypertension owning an HBPM [15, 16]. Previous research on the TIME study found that participants with diabetes mellitus and current smokers were less likely to report owning an HBPM [17, 18]. Besides ownership, recognising factors that influence active participation and long-term commitment to BP self-monitoring may help tailor future HBPM interventions in the community.

In this study, we aim to identify participant-level demographics, co-morbidities, socioeconomic factors and HBPM characteristics that influence active participation (defined as submitting at least one weekly set of BP measurements) and longer-term commitment (defined as submitting at least six sets of BP measurements) to home BP self-monitoring in the TIME study.

## Methods

### Study design and participants

Patients were eligible for enrolment in the TIME study if they were aged 18 or over, receiving treatment for hypertension with one or more antihypertensive drugs, and had a valid email address. Potential study participants were enrolled in the study via a secure website (http://www.timestudy.co.uk). Further details of the TIME study are described in the published study protocol [13]. Participants who reported owning an HBPM were invited to submit home BP measurements regularly throughout the study. All types and brands of personal HBPMs were accepted.

The TIME study is a registered clinical trial (EudraCT 2011-001968-21, ISRCTN18157641) with ethical approval (East of Scotland Research Ethics Service 11/AL/0309).

### Data collection

Baseline data collection in TIME included self-reported demographics, blood pressure-lowering medication(s), and the presence of other co-morbid conditions including angina, myocardial infarction (MI), chronic obstructive pulmonary disease (COPD), impaired kidney function, peripheral vascular disease, arthritis, and cerebrovascular disease (transient ischaemic attack or stroke), and Index of Multiple Deprivation (IMD). The indices of multiple deprivation for England [19], Scotland [20], Wales [21], and Northern Ireland [22] are area-based measures of relative socio-economic deprivation. Postcode areas are ranked from most deprived to least deprived. This ranking is then categorized into ten “deciles” (from most deprived = 1 to least deprived = 10). Individual IMD decile score was derived from residential postcodes using publicly available deprivation data from the different constituent countries and then aggregated into a combined IMD decile score. The TIME Study recruited participants via numerous outreach strategies: advertisements, invitations from general practitioners’ (GP), hospital clinics, and research study databases, etc. [23]. Participants were asked to provide information on how they heard about the study.

An online form asked participants to identify the blood pressure monitor that they owned. A combination of a drop-down menu and a free-text field were available for participants to input the brand, manufacturer and model of their HBPM. HBPMs were categorised by site (upper arm or wrist cuff), validation status (listed as validated according to the British and Irish Hypertension Society (BIHS) or by dabl Educational Trust) and retail price (protocol as described in [18]). HBPMs of participants who indicated they owned HBPMs but did not provide any further information were classified as ‘unknown’.

Participants were provided with a detailed set of instructions on correctly taking BP measurements using their own HBPM, following NICE Guidelines [24]. Participants were instructed to take BP measurements in the sitting position after a minimum of at least five minutes rest. A series of three consecutive measurements in the morning and in the evening, continued for at least four days, and ideally, up to seven days were requested. During the follow-up period, participants were reminded via an automated email to submit a set of home BP measurements at one week, four weeks, twelve weeks, six months and every three months thereafter. For this study, participant follow-up was censored three months after the last known BP measurement before 1st May 2019.

All participant-identifiable data was securely stored on the TIME study database. Data were anonymised before extraction for analysis.

### Outcomes

The two primary outcomes used to evaluate the self-monitoring BP regime were active participation and long-term commitment. Active participation was defined as submitting at least one 4–7 day set of BP measurements after consent to participate. Long-term commitment to the BP self-monitoring protocol was defined as submitting at least six 4–7 day sets of BP measurements post-consent, equivalent to 1 year of active participation.

### Statistical analysis

Continuous data were described using means ± standard deviation (SD) while categorical data were summarised by counts and percentages of the total. Due to participant error in data reporting for BMI variable captured via text-entry (calculated from height and weight) on the online form, outlying data points for BMI were excluded from the analysis. Shapiro-Wilk tests were performed to test for data normality before excluding extreme data points (0.5% of either end of the distribution). A total of 105 extreme data points for BMI were excluded. Multiple imputation was used to impute missing values for age, BMI and socioeconomic status (IMD decile).

A logistic regression model was constructed to determine which factors were associated with active participation. Model variables included age, sex, country (within the UK), smoking status, BMI, comorbidities, family history of hypertension, number of antihypertensive medications, socioeconomic deprivation (IMD decile), recruitment strategy, and HBPM validation status. A second multivariable logistic regression model was constructed to ascertain the effects of the same variables and baseline systolic home blood pressure measurement on the likelihood of long-term commitment to BP-self monitoring.

Due to the unstructured nature of free-text entries of BP measurements from participants, several entries were either implausible or incomplete. For example, some participants may have only submitted 2 days of BP measurements over a 7-day period. We hypothesized that participants who submitted incomplete or implausible data may be sufficiently different from those who submitted complete data to mask associations in the model. A sensitivity analysis was therefore undertaken to assess if the quality of submitted BP measurements would affect output from the logistic regression model. For each participant, aberrant BP values were identified according to the following criteria, previously defined by Bobrie et al. [25]: (a) diastolic blood pressure (DBP) less than 40 mm Hg or greater than 150 mm Hg, (b) systolic blood pressure (SBP) less than 60 mm Hg or greater than 250 mm Hg, or (c) pulse pressure less than 10 mm Hg. Home BP measurements were considered valid and complete if:

1. At least two consecutive BP measurements were taken for each BP recording
2. At least four days of both morning and evening BP measurements were provided over seven days.

The logistic regression model was repeated after excluding BP measurements that did not satisfy these predefined criteria.

All statistical analyses were performed using R version 3.5.0 and RStudio version 1.2.5 (RStudio Inc. Massachusetts, USA). *P*-values < 0.05 were considered statistically significant.

## Results

### Demographics

A total of 21,104 participants were randomized in the TIME study. Of these, 11,059 (52.4%) agreed to submit HBPM measurements and were included in this analysis. The characteristics of these participants are summarized in Table 1. Most of the participants were from England (*n* = 9,919; 89.7%). Remaining participants were from Scotland (*n* = 782; 7.1%), Wales (*n* = 355; 3.2%), and Northern Ireland (*n* = 3; 0.03%). The mean age of the participants randomized was 67.8 ± 8.8 years, of whom 6799 (61.5%) were male, and 5562 (50.3%) were obese (BMI ≥ 30 kg/m^2^). 4579 (41.4%) reported a current or past history of smoking. Diabetes (11.1%), arthritis (9.3%), and stroke/TIA (6.2%) were the most frequently reported co-morbidities. The type of HBPM owned could not be interpreted and identified based on information entered by the participant for 3820 (34.5%) participants.

**Table 1:** Characteristics of study population enrolled into home BP self-monitoring in the TIME study.

Characteristic	Active participation in BP self-monitoring study	
	No	Yes
	(*N* = 3413)	(*N* = 7646)
**Age (years) – mean** ± **SD**	66.3 ± 9.7	68.5 ± 8.3
**Sex – no. (%)**		
Male	2082 (61.0)	4717 (61.7)
Female	1331 (39.0)	2929 (38.3)
**Country – no. (%)**		
England	3089 (90.5)	6830 (89.3)
Scotland	209 (6.1)	573 (7.5)
Wales	113 (3.3)	242 (2.3)
Northern Ireland	2 (0.1)	1 (0)
**Body-mass index (kg/m**^**2**^) **– mean** ± **SD**	31.4 ± 5.9	30.7 ± 5.6
**Smoking status – no. (%)**		
Non-smoker	1934 (56.7)	4498 (58.8)
Current smoker	167 (4.9)	205 (2.7)
Ex-smoker	1301 (38.1)	2906 (38.0)
Unknown	11 (0.3)	37 (0.5)
**Co-morbidities – no. (%)**		
Diabetes	388 (11.4)	845 (11.1)
Angina	119 (3.5)	272 (3.6)
COPD	99 (2.9)	190 (2.5)
Impaired kidney function	121 (3.5)	250 (3.3)
Arthritis	291 (8.5)	734 (9.6)
Peripheral vascular disease	53 (1.6)	91 (1.2)
Myocardial Infarction	157 (4.6)	314 (4.1)
Stroke/TIA	219 (6.4)	467 (6.1)
**Family History of HTN – no. (%)**	2049 (60.0)	4581 (59.9)
**No. of antihypertensive medications – no. (%)**		
1 medication	1939 (56.8)	4016 (52.5)
>2 medications	1408 (41.3)	3464 (45.3)
Unknown	66 (1.9)	166 (2.2)
**Socioeconomic deprivation – no. (%)**		
More deprived (IMD Decile 1–5)	963 (28.2)	1816 (23.8)
Less deprived (IMD Decile 6–10)	2415 (70.8)	5749 (75.2)
Unknown	35 (1.0)	81 (1.1)
**Recruitment method – no. (%)**		
GP	2626 (76.9)	5505 (72.0)
Health Registries (UK Biobank, GoShare)	474 (13.9)	1413 (18.5)
Hospital/Clinic	105 (3.1)	223 (2.9)
Advertisements	75 (2.2)	214 (2.8)
**Characteristics of home BP monitors used**		
**Cuff Site – no. (%)**		
Wrist	139 (4.1)	346 (4.5)
Upper arm	1984 (58.1)	4770 (62.4)
Unknown	1290 (37.8)	2530 (33.1)
**Price – no. (%)**		
≤ than median (£45.00)	1254 (36.7)	2928 (38.3)
> than median (£45.00)	762 (22.3)	1914 (25.0)
Price not found	107 (3.1)	274 (3.6)
Unknown	1290 (37.8)	2530 (33.1)
**Validation Status – no. (%)**		
Non-validated BP monitor	374 (11.0)	991 (13.0)
Validated BP monitor	1749 (51.2)	4125 (53.9)
Unknown	1290 (37.8)	2530 (33.1)

*BP* Blood pressure, *COPD* Chronic obstructive pulmonary disease, *HTN* Hypertension, *IMD* Index of multiple deprivation, *TIA* Transient ischaemic attack.

### Factors influencing active participation in home BP self-monitoring

**Fig. 1:**
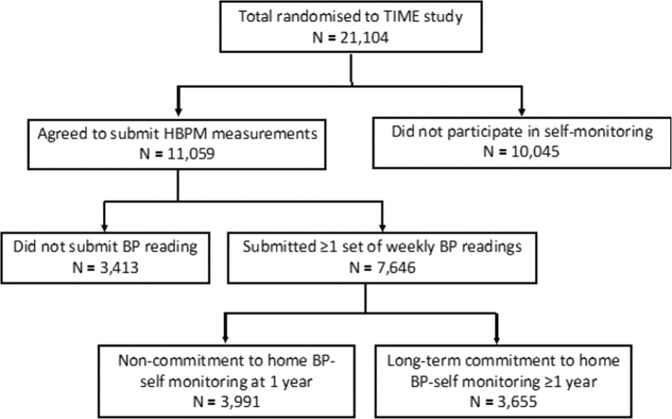
Consort diagram. BP Blood pressure, TIME Treatment in Morning versus Evening, HBPM Home blood pressure monitor.

Of the 11,059 participants who agreed to submit HBPM measurements, only 7646 (69.1%) had submitted at least one blood pressure measurement at the time of analysis (Fig. 1). Participants were found to be more likely to actively participate if they were older (adjusted odds ratio (AOR) per decade, 1.29; 95% CI 1.23–1.36), had a positive family history of hypertension (AOR, 1.11; 95% CI 1.01–1.21), reported more antihypertensive medications (AOR, 1.10; 95% CI 1.04–1.16), or were living in less deprived areas (IMD (per decile): AOR, 1.03; 95% CI 1.01–1.05), using multivariable logistic regression (Table 2). Conversely, higher BMI (AOR per 5.0 kg/m^2^, 0.91; 95% CI 0.88–0.95), active smoking (AOR, 0.63; 95% CI 0.51–0.78) and ownership of an unidentifiable HBPM model (AOR 0.85; 95% CI 0.78–0.93) were negatively associated with active participation. There were no significant differences in these associations when the analysis was stratified by age (under 65, 65 years and over). Interestingly, participants from Scotland (compared to England) and those who heard about the study via email invitations from health registries or advertisements (compared to GP invitations) were more likely to actively participate in BP self-monitoring.

**Table 2:** Logistic regression of factors associated with active participation to home BP self-monitoring in TIME study.

Characteristic	Univariate analysis		Adjusted analysis	
	OR	95% CI	OR	95% CI
Age (per decade)	1.03	1.02–1.03	1.29	1.23–1.36
Sex				
Female	1		1	
Male	1.03	0.95–1.12	1	0.91–1.09
Country				
England	1		1	
Scotland	1.24	1.05–1.46	1.28	1.08–1.51
Wales	0.97	0.77–1.22	0.99	0.79–1.25
Northern Ireland	0.23	0.02–2.49	0.25	0.01–2.66
Body-mass index (per 5.0 kg/m^2^)	0.89	0.86–0.92	0.91	0.88–0.95
Smoking status				
Non-smoker	1		1	
Current smoker	0.53	0.43–0.65	0.63	0.51–0.78
Ex-smoker	0.96	0.88–1.05	0.95	0.87–1.04
Unknown	1.45	0.74–2.84	1.44	0.75–2.99
Comorbidities				
Diabetes	0.97	0.85–1.10	1.02	0.89–1.16
Angina	1.02	0.82–1.28	1.03	0.82–1.31
COPD	0.85	0.67–1.09	0.87	0.67–1.11
Impaired kidney function	0.92	0.74–1.15	0.92	0.73–1.15
Arthritis	1.14	0.99–1.31	1.1	0.95–1.27
Peripheral vascular disease	0.76	0.54–1.08	0.74	0.53–1.07
Myocardial infarction	0.88	0.73–1.08	0.85	0.69–1.05
Stroke/TIA	0.95	0.80–1.12	0.86	0.73–1.02
Family history of HTN	1.06	0.97–1.16	1.11	1.01–1.21
No. of antihypertensive medications	1.08	1.03–1.14	1.1	1.04–1.16
Socioeconomic status				
IMD (per decile)	1.04	1.03–1.06	1.03	1.01–1.05
Recruitment method				
GP	1		1	
Health registries (UK Biobank, GoShare)	1.42	1.27–1.59	1.38	1.23–1.55
Hospital/Clinic	1.01	0.80–1.29	1.14	0.90–1.46
Advertisements	1.36	1.04–1.79	1.38	1.06–1.82
Others	1.04	0.85–1.29	1.06	0.86–1.32
**Characteristics of home BP monitors used**				
Validation status				
Validated BP monitor	1		1	
Non-Validated BP monitor	1.12	0.99–1.28	1.1	0.97–1.26
Unknown	0.83	0.76–0.91	0.85	0.78–0.93

*BP* Blood pressure, *COPD* Chronic obstructive pulmonary disease, *HTN* Hypertension, *IMD* Index of multiple deprivation, *TIA* Transient ischaemic attack.

### Factors influencing long-term commitment to home BP self-monitoring

After one year, only 3655 (47.8%) participants were still actively providing BP measurements, defined previously as submitting at least six 4–7 day sets of BP measurements (Fig. 1). Continued submission of BP measurements beyond one year was significantly associated with increased age at baseline (AOR per decade, 1.26; 95% CI 1.19–1.34) and positive family history of hypertension (AOR, 1.14, 95% CI 1.03–1.26) in the regression analysis (Table 3). Participants residing in less deprived socioeconomic regions, or recruited via health registries or advertisements, were more likely to exhibit a long-term commitment to BP self-monitoring in the study. Higher BMI (AOR per 5.0 kg/m^2^, 0.88, 95% CI 0.85–0.92), current smoking (AOR, 0.58, 95% CI 0.43–0.79) and higher systolic pressure at baseline (AOR per 1 mm Hg, 0.99, 95% CI 0.98–0.99) were negatively associated with commitment beyond one year of enrolment. There were no significant differences in these associations when the analysis was stratified by age (under 65, 65 years, and over). Owning an HBPM without validation evidence was associated with a reduced likelihood of continued submission of BP measurements (AOR, 0.83; 95% CI 0.75–0.91) (Table 3).

**Table 3:** Logistic regression of factors associated with long-term commitment to home BP self-monitoring in the TIME study.

Subgroup	Continued submission of BP measurements beyond 1 year		Adjusted analysis	
	Yes (%)	No (%)	OR	95% CI
	*N* = 3655	*N* = 3991		
**Participant Characteristics**				
Age, mean (SD)	69.3 (7.3)	67.7 (9.0)	1.29	1.21–1.37
			(per decade increase)	
Sex				
Female	1410 (38.6)	1519 (38.1)	1	
Male	2245 (61.4)	2472 (61.9)	0.98	0.89–1.08
Country				
England	3244 (88.8)	3586 (89.9)	1	
Scotland	292 (8.0)	281 (7.0)	1.16	0.97–1.38
Wales	119 (3.3)	123 (3.1)	1.07	0.82–1.40
Body-mass index	30.2 (5.3)	31.1 (5.6)	0.89	0.85–0.93
			(per 5.0 kg/m^2^ increase)	
Smoking status				
Non-smoker	2190 (59.9)	2308 (57.8)	1	
Current smoker	66 (1.8)	139 (3.5)	0.6	0.44–0.82
Ex-smoker	1383 (37.8)	1523 (38.2)	0.97	0.88–1.08
Unknown	16 (0.4)	21 (0.5)	0.85	0.43–1.65
Comorbidities				
Diabetes	386 (10.6)	459 (11.5)	1	0.86–1.15
Angina	118 (3.2)	154 (3.9)	0.84	0.65–1.09
COPD	79 (2.2)	111 (2.8)	0.83	0.61–1.12
Impaired kidney function	119 (3.3)	131 (3.3)	1	0.77–1.29
Arthritis	344 (9.4)	390 (9.8)	0.95	0.81–1.12
Peripheral vascular disease	38 (1.0)	53 (1.3)	0.85	0.55–1.31
Myocardial infarction	144 (3.9)	170 (4.3)	0.94	0.74–1.19
Stroke/TIA	212 (5.8)	255 (6.4)	0.83	0.69–1.01
Family History of HTN				
No	1046 (28.6)	1231 (30.8)	1	
Yes	2467 (67.5)	2635 (66.0)	1.15	1.03–1.27
No. of antihypertensive medications	1.57 (0.8)	1.60 (0.8)	1	0.94–1.06
Socioeconomic status				
IMD, mean decile (SD)	7.32 (2.3)	7.11 (2.5)	1.02	1.00–1.04
Recruitment method				
GP	2480 (67.9)	3025 (75.8)	1	
Health Registries (UK Biobank, GoShare)	797 (21.8)	616 (15.4)	1.53	1.36–1.73
Hospital/Clinic	104 (2.8)	119 (3.0)	1.14	0.87–1.50
Advertisements	131 (3.6)	83 (2.1)	1.93	1.46–2.58
Others	143 (3.9)	148 (3.7)	1.18	0.92–1.50
**HBPM Characteristics**				
Baseline systolic blood pressure measurement, mean (SD)	131.2 (9.6)	132.3 (11.3)	0.99	0.98–0.99
Validation status of HBPM				
Yes	2043 (55.9)	2082 (52.2)	1	
No	498 (13.6)	493 (12.4)	1.03	0.89–1.19
Unknown	1114 (30.5)	1416 (35.5)	0.81	0.73–0.90

*HTN* Hypertension, *IMD* Index of multiple deprivation.

### Sensitivity analysis for quality of submitted BP measurements

After excluding 353 participants who did not submit BP measurements that satisfied the predefined validity criteria, 7293 participants had submitted at least one set of measurements. 3428 (47.0%) were still actively providing BP measurements beyond one year. When limiting the analysis to participants who submitted valid sets of BP measurements only, positive family history of hypertension (AOR, 1.09; 95% CI 0.98–1.21) and socioeconomic status (AOR per decile, 1.02; 95% CI 0.99–1.04) were not statistically significantly associated with long-term commitment, although point estimates favoured continued submission of BP measurements beyond one year.

## Discussion

### Principal findings

This study provides valuable insights into the medical, social and economic factors associated with active participation in, and long-term commitment to, HBPM measurement in a large UK remote clinical trial by participants with hypertension. Increased age, a positive family history of hypertension, a higher number of antihypertensive medications, and higher socioeconomic status were associated with active participation in the study. Current smokers and people with increased BMI were less likely to participate actively. Participants with non-modifiable risk factors for hypertension – increased age and positive family history of hypertension – were also more likely to retain interest and continue submitting BP measurements one year from enrolment. In contrast, participants with higher BMI, active smokers or who had higher systolic pressures at baseline were less likely to submit BP measurements beyond the first year. TIME Participants recruited via health registries or advertisements were more likely to actively participate and maintain engagement in BP self-monitoring.

### Meaning of the study

Longitudinal studies are necessary to identify patterns or changes over time. However, more extensive follow-up periods are associated with time-dependent increasing risk of attrition [26]. In this study, continued participation in BP self-monitoring at 1-year was 46.2%. Younger age was found to be associated with lower long-term commitment. Similarly, in a longitudinal population-based survey of women in Australia, the attrition rate was higher amongst younger women than middle and older-aged women [27]. While participants did not actively withdraw from the TIME study, some were not sufficiently motivated to continue submitting HBPM-derived measurements. The term “inclined abstainers” has been suggested to describe such participants, where positive intentions to engage at the start of the study are not translated to sustained action [28]. Despite the potential benefits of the remote study design in reducing the burden of face-to-face study visits, non-participation and attrition rates for home blood pressure monitoring were still high. Factors influencing retention in remote studies may be different from those influencing retention in more traditional site-based clinical trials. Further work will be needed to understand this better as remote trials become more common.

The finding that individuals with modifiable cardiovascular risk factors (smoking, obesity, and higher baseline systolic blood pressure) are less likely to participate actively or continue engaging in home BP monitoring beyond one-year warrants further attention. Other studies have also seen a similar association, and it has been proposed that more risky lifestyle behaviour is associated with non-adherence with self-monitoring and interventions [29, 30]. As younger participants, and those with established modifiable risk factors, are at increased lifetime risk of cardiovascular events compared to the general population with hypertension, implementation of interventions involving self-monitoring of blood pressure should be accompanied by consideration of additional efforts to engage them. These efforts could reduce the long-term negative burden of hypertension and its associated complications and their impact on the National Health Service finances and resource allocation.

Socioeconomic deprivation may further complicate the widespread implementation of self-monitoring of hypertension. We found that residence in less deprived postcode areas was associated, albeit to a small degree, with increased participation and continued engagement in home BP self-monitoring. However, low representation of participants from deprived areas in the TIME study (6.2% from IMD decile 1 and 2 vs. 33.9% IMD decile 9 and 10) and lower self-reported ownership of HBPMs amongst deprived individuals (44.4% from IMD decile 1 and 2 vs. 58.0% IMD decile 9 and 10) may mask true socioeconomic differences in self-monitoring of blood pressure. For this group of patients, additional support may be needed.

A detailed analysis of the types and validation statuses of HBPMs owned by participants in the TIME Study has been previously described [18]. Concerning only participants who owned identifiable HBPMs (*n* = 7239), 81.1% of participants owned a device validated by BIHS or the dabl Education Trust. The association between unidentifiable HBPM devices (34.5% of participants) and reduced participation and long-term commitment to home BP self-monitoring is concerning. This finding further reinforces the need to educate people with hypertension on the importance of incorporating a device’s validation status into decision-making when choosing an HBPM. Possible reasons that HBPM devices were unidentifiable include (1) errors in participant reporting (inputting vendor’s name, brand name with no identifiers of a specific model), (2) inability to find relevant information on the device, or (3) using an unbranded home BP monitor.

### Strengths and limitations

Participation in self-monitoring of BP in the TIME study was voluntary; this mirrors everyday HBPM use in clinical care. However, it is not clear that our findings are directly applicable to the general population with hypertension. The study investigators had limited direct communication with participants; only one reminder email was sent to participants to submit BP measurements regularly. However, again, this light-touch direction is similar to the clinical use of home BP monitoring. These pragmatic features of the TIME study mean that it is more likely that the findings reported here may be externally valid.

Our study has some notable limitations. This study comprised voluntary study participants, a group more likely to exhibit greater responsibility in their day-to-day chronic disease management. We hypothesize that the degree of active participation in a real-world setting may be much lower, particularly considering that only a third of people with hypertension currently own an HBPM [15, 16]. The reliance on participant-reported data – baseline characteristics, information on the HBPM model and longitudinal BP measurements – was necessary to achieve the efficiency and cost-effectiveness of undertaking a remote study of this scale. It does, however, mean that routine validation of these data was not possible. Although the TIME study has participants from across the UK, the very small numbers from Northern Ireland mean that conclusions about country-specific effects may not be generalizable to Northern Ireland.

Finally, we used the best sources of HBPM validation status available at the time of analysis, dabl and BIHS. The STRIDE BP website (www.stridebp.org), since launching in 2019, now provides an extensive clinically and scientifically supported HBPM validation status list; this will be an important source of such information for future similar research [31].

### Future research and clinical practice

The costs of an ageing population, rising numbers of people with long term medical conditions, and the current coronavirus pandemic are all driving the introduction of telemonitoring in the UK [32]. Scale-up BP is a project funded by the Scottish Government to assess the feasibility of implementing telemonitoring of blood pressure at scale for patients with hypertension in primary care [33, 34]. Stoddart et al. showed that supported telemonitoring of BP was more effective at reducing BP than standard care but was also significantly more expensive [35]. Results from our study could be used to inform the allocation of resources in such projects. Individuals at risk of non-participation or drop-out, e.g., those with modifiable risk factors for cardiovascular disease, might benefit from increased support. This could reduce loss to follow-up and improve the cost-effectiveness of BP programmes.

## Conclusion

A willingness to monitor one’s condition is a prerequisite for effective self-management. In a large study of participants in a clinical trial of hypertension in the UK, we have shown that individuals with non-modifiable risk factors (older age and family history of hypertension) were likely to participate actively and remain committed in the longer-term to home blood pressure monitoring. Conversely, participants with higher BMI, current smokers or higher systolic blood pressures at baseline were more likely to stop submitting BP data to the study. Insights gained from this study should allow future researchers and clinicians to effectively target follow-up strategies and minimise loss to follow-up in usual healthcare settings and remote clinical trials.

### Summary table

#### What is known about this topic?

- Elevated home blood pressure measurements are strongly correlated with long-term complications of hypertension.
- Supported home blood pressure monitoring can be effectively used in hypertension management.

#### What this study adds?

- In a pragmatic clinical trial, patient-level characteristics affect the likelihood of active and sustained participation in home blood pressure monitoring using participant-owned monitors.
- Younger people and those with modifiable cardiovascular risk factors are less likely to remain engaged with home blood pressure monitoring after one year.
